# Estimation of the lateral variation of photon beam energy spectra using the percentage depth dose reconstruction method

**DOI:** 10.1007/s12194-024-00835-5

**Published:** 2024-09-06

**Authors:** Puspen Chakraborty, Hidetoshi Saitoh, Yuta Miyake, Tenyoh Suzuki, Weishan Chang

**Affiliations:** 1 Department of Radiological Sciences, Graduate School of Human Health Sciences, Tokyo Metropolitan University, Tokyo, Japan; 2 Department of Application Physics, Elekta K.K., Tokyo, Japan

**Keywords:** Lateral energy spectrum variation, Off-axis softening, Photon-collapsed cone convolution, Beam modeling

## Abstract

In photon-collapsed cone convolution (pCCC) algorithm of the Monaco treatment planning system (TPS), the central-axis energy spectrum is assumed constant throughout the entire irradiation area. To consider lateral variations, an off-axis softening factor is applied to attenuation coefficients during the total energy released per unit mass calculation. We evaluated this method through comparison studies of percentage depth doses (PDDs) and off-axis ratios (OARs) calculated by Monaco and measured for a 6 MV photon beam at various off-axis angles and depths. Significant differences were observed, with relative differences exceeding ± 1%. Therefore, this method may not accurately represent lateral variations of energy spectra. We propose directly implementing energy spectra on both central-axis and off-axis to improve dose calculation accuracy for large field. To this end, we introduce reconstruction of PDDs from monoenergetic depth doses (MDDs) along off-axis angles, thereby estimating energy spectra as functions of radial distance. This method derives energy spectra quickly without significantly increasing the beam modeling time. MDDs were computed through Monte Carlo simulations (DOSRZnrc). The variances between reconstructed and measured PDDs were minimized using the generalized-reduced-gradient method to optimize energy spectra. Reconstructed PDDs along off-axis angles of 0°, 1.15°, 2.29°, 3.43°, 4.57°, 5.71°, 6.84°, 7.97°, 9.09°, 10.2° to estimate energy spectra at radial distances of 0–18 cm in 2 cm increments and OARs calculated using estimated energy spectra at 5, 10, and 20 cm depths, well agreed with measurement (relative differences within ± 0.5%). In conclusion, our proposed method accurately estimates lateral energy spectrum variation, thereby improving dose calculation accuracy of pCCC algorithm.

**Supplementary Information:**

The online version contains supplementary material available at 10.1007/s12194-024-00835-5.

## Introduction

The accuracy of the photon-collapsed cone convolution (pCCC) algorithm strongly depends on the agreement between the estimated and actual energy spectrum. Several methods have been proposed to estimate energy spectrum through measurements or Monte Carlo (MC) simulation. Measurements can be directly performed using a NaI (Tl) scintillator at ultra-low dose rates [1], which might be limited to certain linear accelerators (linac), or indirectly performed through scattered-photon detection using Compton spectroscopy, which requires large amounts of lead shielding and a high setting accuracy [2, 3]. The most reliable approach is MC simulation of a precisely modeled treatment head, which requires a time-consuming process to define the energy and spatial distributions of the initial electrons incident on the target. Moreover, to model different energies or linacs, even from the same manufacturer, the entire procedure must be repeated [4–6]. Sauer et al. [7] proposed a numerical percentage depth dose (PDD) reconstruction method that quickly derives the energy spectrum for the convolution algorithm while avoiding experimental complexities. This method reconstructs PDD in agreement with measured PDD by employing weighted superpositions of monoenergetic depth doses (MDDs). The PDDs are matched using the least squares method, with weights determined through the Wolfe-reduced-gradient method [8]. The resultant weights are interpreted as discrete energy fluences. However, several constraints must be imposed to construct a realistic energy spectrum. In contrast, Ahnesjo et al. [9] simplified the study of Andreo and Brahme [10] and proposed an analytical model that reconstructs PDD by estimating the energy spectrum. This model requires four parameters: the mean energy of the initial electrons, the target and flattening filter thicknesses, and the effective atomic number. By reconstructing PDD from MDDs in agreement with measured PDD, parameters are determined, and thus, deriving energy spectrum. The energy spectrum estimated by Ahnesjo et al. [9] is initially applied in the beam modeling of pCCC algorithm of the Monaco [Elekta AB, Stockholm, Sweden] treatment planning system (TPS) and adjusted to match the calculated and measured PDD.

The PDD reconstruction methods adopted in previous studies [7, 9] estimate the energy spectrum only at the central-axis. It has been reported that mean energies of 6 MV and 15 MV photon beams are reduced by approximately 22% and 25%, respectively, at an off-axis distance of 18 cm on a typical linac [4, 6, 11, 12]. These variations have been primarily attributed to off-axis softening of the energy spectrum, which depends on the geometry and composition of the flattening filter. Therefore, determining the energy spectra as functions of distance from the central-axis is essential for accurate dose calculation [4, 13, 14], especially for a large-field irradiation such as esophageal cancer with cervical lymph-node metastasis or cervical cancer with para-aortic lymph-node metastasis. To simplify the beam modeling process, Monaco TPS assumes a constant central-axis energy spectrum throughout the entire irradiation area. In the pCCC algorithm, dose calculation is performed by convolving the point-energy deposition kernel with the total energy released per unit mass (TERMA). As the energy spectrum is constant, off-axis softening with the depth-hardening are applied to the attenuation coefficients during the TERMA calculation [15]. The off-axis softening factor is obtained from a third-degree polynomial fit of the half value layers (identical to the attenuation coefficients) given by Taylor et al. [15], where the fitting parameters were demonstrated to be independent of beam energies and linacs. The depth-hardening factor is determined by fitting the graph of hardening factors as a function of effective photon energy provided by Yu et al. [11]. The point-energy deposition kernel, which is less sensitive to energy spectral variation than TERMA [16], is calculated for the TERMA-weighted spectrum at a depth of 10 cm. However, as the accuracy of this off-axis softening process has not been investigated in detail, the sufficiency of precisely modeling the off-axis energy spectral distribution is doubtful.

The present study aims to accurately estimate energy spectra as functions of radial distance on the isocenter plane by reconstructing PDDs at various off-axis angles. This approach intends to improve the dose calculation accuracy of the pCCC algorithm (Monaco TPS) for large fields. Although the off-axis ratio (OAR) is a common measurement in radiation dosimetry, it mainly reflects the photon fluence distribution [5]. Therefore, we utilized PDDs in angular directions to estimate the lateral variation of energy spectra. The purpose of using PDD reconstruction method was to derive energy spectra within a short time, ensuring that the beam modeling time does not increase. Meanwhile, the accuracy of the aforementioned off-axis softening process of the Monaco TPS was investigated by analyzing both PDDs and OARs.

## Methods

### Theory of the PDD reconstruction method

The PDD in a water phantom at depth *d* along an off-axis angle *θ* for a field size ${A}_{0}$ irradiated with a continuous energy spectrum $\lambda$, can be calculated as follows:

1 $$\begin{array}{c} PDD \left(\lambda , {A}_{0}, d, \theta \right)= {\large\frac{\underset{{E}_{\text{min}}}{\overset{{E}_{\text{max}}}{\int }} {{\varPhi }}_{E}(\theta )\,\times \,D \left(E, \,{A}_{0}, \,d, \,\theta \right) \text{d}E}{\underset{{E}_{\text{min}}}{\overset{{E}_{\text{max}}}{\int }}{{\varPhi }}_{E}(\theta )\,\times \,D \left(E, \,{A}_{0}, \,{d}_{\text{max}}, \,\theta \right) \text{d}E}} \times 100, \end{array}$$

where ${\varPhi}_{E}(\theta )$ is the differential photon fluence and $D(E, {A}_{0}, d, \theta )$ is the absorbed dose per photon of monochromatic energy *E*. Since the source-surface distance (SSD) varies with *θ*, it has been included as an influencing parameter of $D(E, {A}_{0}, d, \theta )$. *d* is considered beyond the depth of maximum dose *d*_max_ due to significant dose contributions from the charged-particles in the build-up region. As the PDD varies slightly within a small energy range, depending on the properties of the mass attenuation coefficient, the energy spectrum can be handled as a discrete probability distribution [7, 9] and Eq. (1) can be rewritten as follows:

2 $$\begin{array}{c}PDD \left(\lambda , {A}_{0}, d, \theta \right)={\large\frac{\sum_{i = 1}^{n}{p}_{i}\left(\theta \right)\,\times \,D \left({E}_{i},\,{A}_{0},\,d, \,\theta \right)\Delta E}{\sum_{i = 1}^{n}{p}_{i}\left(\theta \right)\,\times \,D \left({E}_{i},\,{A}_{0},\,{d}_{\text{max}}, \,\theta \right)\Delta E}} \times 100,\end{array}$$

where *n* is the total number of energy bins *i* of the discrete energy interval $\Delta E$ and ${E}_{i}$ is the mid-energy of each energy bin. The relative fluence ${p}_{i}(\theta )$ can be defined as follows:

3 $$\begin{array}{c}{p}_{i}\left(\theta \right)= {\large\frac{ {{\{\varPhi}_{E}\left(\theta \right)\,\times\,\Delta E\}}_{i}}{{\sum }_{i=1}^{n}{{\{\varPhi}_{E}\left(\theta \right)\,\times\,\Delta E\}}_{i}}},\end{array}$$

where $\Delta {E}_{i}$ is the width of the *i*th energy bin. ${p}_{i}(\theta )$ can be obtained by minimizing the variance *σ*^2^ of the relative difference *δ*_*j*_ (%) between the reconstructed and measured PDDs. In this study, *σ*^2^ was calculated as

4 $$\begin{array}{c}{\sigma }^{2} = {\large\frac{1}{m}}\sum_{j = 1}^{m}{{\delta }_{j}}^{2}= {\large\frac{1}{m}}\sum_{j = 1}^{m}{{\left({\large\frac{{PDD}_{\text{recons}} - {PDD}_{\text{meas}}}{{PDD}_{\text{meas}}}} \times 100\right)}_{j}}^{2},\end{array}$$

where *m* is the total number of sampling depths *j*. The optimization process employed the generalized-reduced-gradient (GRG), which generalizes the reduced gradient method by allowing nonlinear constraints and arbitrary bounds on the variables [17]. As mentioned above, the PDD varies slightly within small energy range, so the GRG can generate unrealistic energy spectrum. Therefore, several constraints were applied to obtain a realistically shaped energy spectrum (i.e., ${p}_{i - 1}\le {p}_{i}$ in the energy range lower than energy ${E}_{\text{p}}$ where the fluence is maximum and ${p}_{i}\ge {p}_{i+1}$ in the energy range higher than ${E}_{\text{p}}$) [7]. Similarly, to ensure the gradual off-axis softening of energy spectra from the central-axis to the maximum radial distance, the following constraints were imposed:

$${\left({p}_{i - 1}\right)}_{0 \text{cm}}\le {\left({p}_{i - 1}\right)}_{2 \text{cm}}\le { \left({p}_{i-1}\right)}_{4 \text{cm}}\cdot \cdot \cdot \cdot \le {\left({p}_{i - 1}\right)}_{18 \text{cm }}[\text{while} \space{} {E}_{i}<{E}_{\text{p}}]$$

5 $$\begin{array}{c}{\left({p}_{i + 1}\right)}_{0\text{ cm}}\ge {\left({p}_{i + 1}\right)}_{2 \text{cm}}\ge {\left({p}_{i + 1}\right)}_{4 \text{cm}}\cdot \cdot \cdot \cdot \ge {\left({p}_{i + 1}\right)}_{18 \text{cm}} \left[\text{while} \space{} {E}_{i}>{E}_{\text{p}}\right].\end{array}$$

Here, the numeric subscripts indicate radial distance ${r}_{0}$ on the surface of the water phantom. In addition, to satisfy the condition of relative fluence according to Eq. (3), the constraint $\sum_{i = 1}^{n}{p}_{i}(\theta )=1$ was applied. In the pCCC algorithm of the Monaco TPS, energy fluence ${\varPsi }_{i}$ is applied for the TERMA calculation [15, 18]. Therefore, ${p}_{i}(\theta )$ was converted to ${\varPsi}_{i}(\theta )$ by the following equation:

6 $$\begin{array}{c}{\varPsi}_{i}\left(\theta \right)= {p}_{i}\left(\theta \right)\times {E}_{i}.\end{array}$$

### Measurement of dose distribution

**Fig. 1:**
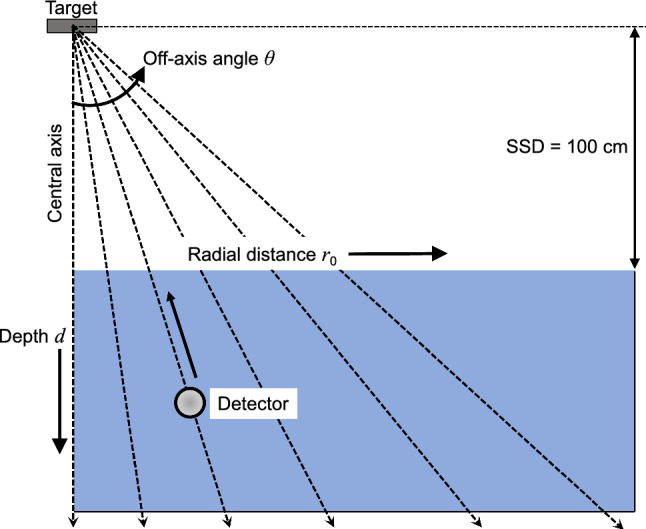
Measurement of percentage depth doses (PDDs) along the off-axis angles *θ* of 0°, 1.15°, 2.29°, 3.43°, 4.57°, 5.71°, 6.84°, 7.97°, 9.09°, and 10.2° for a 6 MV photon beam emitted from a Versa HD linac with a field of 40 cm × 40 cm and a source-surface distance (SSD) of 100 cm

To estimate the energy spectra at radial distance ${r}_{0}$ ranging from 0 to 18 cm in 2 cm increments, the PDDs were measured along the off-axis angles *θ* (${r}_{0}$) of 0° (0 cm), 1.15° (2 cm), 2.29° (4 cm), 3.43° (6 cm), 4.57° (8 cm), 5.71° (10 cm), 6.84° (12 cm), 7.97° (14 cm), 9.09° (16 cm), and 10.2° (18 cm) (see Fig. 1). Measurements were conducted using a 6 MV photon beam emitted from a Versa HD linac [Elekta Oncology Systems, Crawley, UK] with a 40 cm × 40 cm field and an SSD of 100 cm. The blue phantom^2^ with beam-scanning software OmniPro-Accept 7 and CC13 waterproof ionization chambers [IBA dosimetry, GmbH, Germany] were used for the beam-scanning. As the radius of the active chamber cavity was 3.0 mm, the effective point of measurement was set 1.8 mm toward the source from the geometrical center of the field chamber [19]. In the beam-scanning software, the scan type was set to “Fan line” and the PDDs were measured up to depths of 25 cm in 2 mm increments. Although the measurements were performed along off-axis angles, the depths were determined to be parallel to the central-axis to simplify the calculation of MDDs. The PDDs were normalized to the dose at 10 cm depth (*d*_10_) to reduce the effects of charged-particle contamination and to avoid the positional uncertainty of *d*_max_. With the same field size and SSD, the OARs were measured at depths of 5, 10, and 20 cm for verification of the method.

### Estimation of energy spectra

**Fig. 2:**
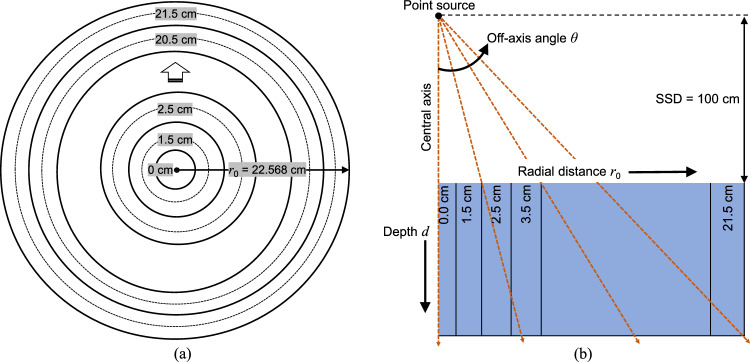
**a** Cylindrical geometry with a circular field of radius 22.568 cm (≡ 40 cm × 40 cm square field) at 100 cm source-surface distance (SSD) to calculate the monoenergetic depth doses (MDDs) along the central-axis and at radial distances ${r}_{0}$ of 1.5 to 21.5 cm in 1.0 cm increments using the DOSRZnrc code; **b** Conversion of MDDs along the off-axis angles *θ* of 0°, 1.15°, 2.29°, 3.43°, 4.57°, 5.71°, 6.84°, 7.97°, 9.09°, and 10.2° using the linear interpolation method

To generate energy spectra for a 6 MV polyenergetic photon beam, the range of monoenergetic photons were considered from 0.05 to 7.00 MeV [1, 6, 14]. As mentioned in Sect. 2.1, the PDD varies slightly within small energy range, depending on the mass attenuation coefficient, which changes rapidly in the low-energy region and slowly in the high-energy region. To optimize the number of energy bins, the widths of the energy bins $\Delta {E}_{i}$ were gradually increased in accordance with the mass attenuation coefficient, defined as follows: 0.05–0.15 MeV, 0.15–0.25 MeV, 0.25–0.35 MeV, 0.35–0.50 MeV, 0.50–0.70 MeV, 0.70–1.00 MeV, 1.00–1.40 MeV, 1.40–1.90 MeV, 1.90–2.50 MeV, 2.50–3.25 MeV, 3.25–4.10 MeV, 4.10–5.15 MeV, 5.15–7.00 MeV. The mid-energy ${E}_{i}$ of these corresponding energy bins were considered for the MDD calculation. MDDs for the energies of 0.100, 0.200, 0.300, 0.425, 0.600, 0.850, 1.200, 1.650, 2.200, 2.875, 3.675, 4.625, and 6.075 MeV were calculated in water along the central-axis and at ${r}_{0}$ of 1.5–21.5 cm in 1.0 cm increments (see Fig. 2a). The MDDs were calculated in cylindrical geometry using DOSRZnrc, a component of the egs_inprz graphical user interface [20], to reduce statistical uncertainty and to derive energy spectra as functions of ${r}_{0}$. The calculations assumed a point source with a circular field of radius 22.568 cm (≡ 40 cm × 40 cm square field) at 100 cm SSD and depths up to 25 cm in 2 mm increments. The electron and photon cut-off energies were set to ECUT = 0.521 MeV and PCUT = 0.01 MeV, respectively. The number of histories per calculation was 2 × 10^9^ except for the central-axis, where it was raised to 3 × 10^9^. A higher number of histories was applied to reduce the statistical uncertainty due to smaller calculation area at the central-axis. To convert MDDs from the cylindrical geometry to the angular directions following the measured PDDs as shown in Fig. 2b, linear interpolation method was applied. Required radial distances $r\left(d\right)$ at depth *d* along the angular directions were calculated as follows:

7 $$\begin{array}{c}r\left(d\right)= {\large\frac{{r}_{0}}{\text{SSD}}} \times \left(\text{SSD}+d\right).\end{array}$$

After formulating a simple mathematical model in Microsoft Excel, varying the MDDs and constraints of the off-axis softening at each ${r}_{0}$, the GRG of the add-in program “Solver” was iterated until the reconstructed PDD acceptably agreed with the measured PDD. Thus, ${p}_{i}(\theta )$ was obtained by minimizing ${\sigma }^{2}$.

### Comparison of estimated energy spectrum at the central-axis with that of Monaco TPS

Energy spectrum at the central-axis was obtained from the commissioning report of the pCCC algorithm (Monaco TPS) and compared with the energy spectrum estimated in this study. For comparison, the energy spectrum of the Monaco TPS was rebinned to match the energy-bin division of the present study. In addition, the mean energy in off-axis angle $\theta$, $\overline{E }\left(\theta \right)$ for both energy spectra was calculated as [21]

8 $$\begin{array}{c}\overline{E }\left(\theta \right)= {\large\frac{\sum_{i=1}^{n}{\varPsi}_{i}(\theta )}{\sum_{i=1}^{n}{p}_{i}(\theta )}}=\sum_{i=1}^{n}{\varPsi}_{i}(\theta ),\end{array}$$

since, $\sum_{i=1}^{n}{p}_{i} (\theta )=1$ as mentioned in Sect. 2.1.

### Comparison of PDDs and OARs

To analyze the off-axis softening of the Monaco TPS, PDDs were computed in water using the pCCC algorithm of the Monaco TPS for a 6 MV photon beam with a 40 cm × 40 cm field and an SSD of 100 cm along the off-axis angles *θ* as mentioned in Sect. 2.2. Calculations were performed on a 50 cm × 50 cm × 40 cm virtual water phantom with a 3 mm grid size. Data were collected using the “Dose plane output” option of the Monaco TPS. The calculated PDDs were compared with both the reconstructed and measured PDDs.

Simultaneously, the OARs were calculated using the Monaco TPS and the estimated energy spectra with their corresponding MDDs at depths of 5, 10, and 20 cm. Fluence ratios required for calculating OARs using the estimated energy spectra were determined by the Microsoft Excel add-in program “Solver” to achieve OARs in agreement with measured OARs. Calculated OARs were compared with measured OARs. The PDD and OAR comparisons were evaluated by the depth-dependent relative difference *δ*_*j*_ (%) and the radial-dependent relative difference *δ*_*r*_ (%), both defined according to Eq. (4).

## Results

**Fig. 3:**
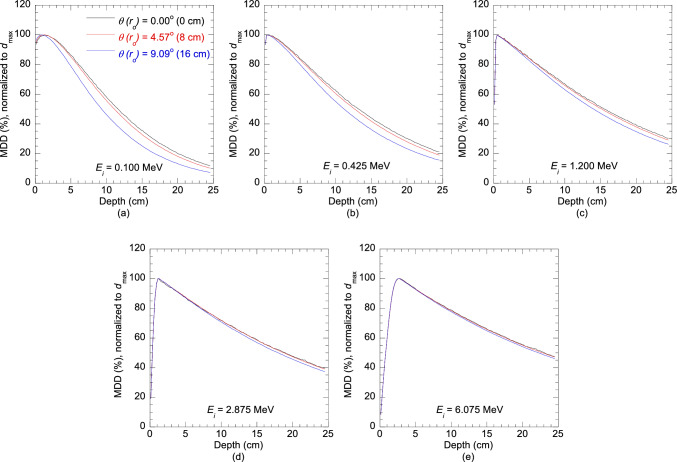
Monoenergetic depth doses (MDDs) [Gy/Incident fluence] for energy *E*_*i*_ of 0.100, 0.425, 1.200, 2.875 and 6.075 MeV along the off-axis angles $\theta$ of 0°, 4.57° and 9.09°, presented as MDDs (%), normalized to the depth of maximum dose *d*_max_

Figure 3 shows the MDDs for energy *E*_*i*_ of 0.100, 0.425, 1.200, 2.875 and 6.075 MeV at radial distances ${r}_{0}$ of 0 cm, 8 cm and 16 cm, along the off-axis angles $\theta$ of 0°, 4.57° and 9.09°. In this figure, MDDs (Gy/Incident fluence) are presented as MDDs (%), normalized to *d*_max_ to show the variation with energy and off-axis angle. Increasing the number of histories from 2 × 10^9^ to 3 × 10^9^ at the central-axis reduced the average standard deviation of the MDDs from ± 0.45% to ± 0.35%. While further increasing the number of histories could reduce this standard deviation even more, it would significantly increase the calculation time. For other ${r}_{0}$, the number of histories was 2 × 10^9^. The average standard deviation at ${r}_{0}$ of 4–16 cm in 4 cm increments were ± 0.15%, ± 0.10%, ± 0.09%, ± 0.08%, respectively. As ${r}_{0}$ increased, so did the calculation area, resulting in a gradual decrease in statistical uncertainties. This suggests that with increasing ${r}_{0}$, the number of histories could be reduced to decrease the calculation time. Detailed data on the MDDs (Gy/Incident fluence) and their corresponding standard deviations is provided in Online Resource 1.

**Fig. 4:**
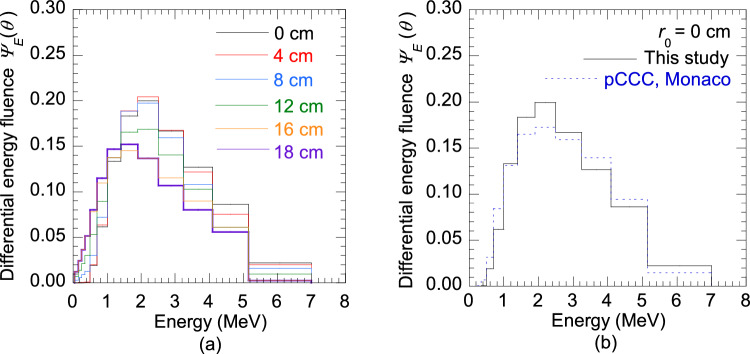
Comparisons of **a** relative energy spectra expressed as differential energy fluence ${\varPsi}_{E}(\theta )$ at radial distances ${r}_{0}$ of 0, 4, 8, 12, 16 and 18 cm and **b** energy spectrum on the central-axis obtained from the commissioning report of the pCCC (Monaco TPS) and estimated in this study

Table 1 presents the relative energy spectra determined by the PDD reconstruction method at ${r}_{0}$ of 0–18 cm in 2 cm increments expressed as energy fluence ${\varPsi}_{i}(\theta )$ of energy interval $\Delta {E}_{i}$. To illustrate the off-axis softening, Fig. 4a compares the estimated energy spectra as differential energy fluence ${\varPsi}_{E}(\theta )$ at ${r}_{0}$ of 0, 4, 8, 12, 16 and 18 cm. As the ${r}_{0}$ increased, a gradual increase in the low-energy fluences and decrease in the high-energy fluences were observed. Figure 4b compares the energy spectra at the central-axis obtained from the commissioning report of the pCCC algorithm (Monaco TPS) and the present study. The energy spectrum of the Monaco TPS was slightly softer than the estimated energy spectrum. The mean energy $\overline{E }\left(\theta \right)$ for energy spectrum of the Monaco TPS was 2.24 MeV, whereas it was 2.31 MeV from estimated energy spectrum.

**Table 1:** Relative energy spectra determined by the PDD reconstruction method at different radial distances ${r}_{0}$ (0 cm to 18 cm) expressed as energy fluence ${ {\varPsi }}_{i}(\theta )$ of energy interval $\Delta {E}_{i}$

Energy *E*_*i*_ (MeV)	Energy fluence ${\varPsi}_{i}(\theta )$ of energy interval $\Delta {E}_{i}$									
	0 cm	2 cm	4 cm	6 cm	8 cm	10 cm	12 cm	14 cm	16 cm	18 cm
0.100	3.745*E *− 05	3.774*E* − 05	7.440*E* − 05	3.757*E* − 04	1.024*E* − 03	1.646*E* − 03	2.339*E* − 03	2.668*E* − 03	3.893*E* − 03	3.867*E* − 03
0.200	7.489*E* − 05	7.552*E* − 05	1.488*E* − 04	7.513*E* − 04	2.047*E* − 03	3.292*E* − 03	4.679*E* − 03	5.335*E* − 03	7.786*E* − 03	7.734*E* − 03
0.300	1.123*E* − 04	1.133*E* − 04	2.232*E* − 04	1.148*E* − 03	3.071*E* − 03	4.938*E* − 03	7.018*E* − 03	8.003*E* − 03	1.168*E* − 02	1.160*E* − 02
0.425	2.387*E* − 04	2.407*E* − 04	4.743*E* − 04	2.440*E* − 03	6.526*E* − 03	1.049*E* − 02	1.491*E* − 02	1.701*E* − 02	2.482*E* − 02	2.465*E* − 02
0.600	1.352*E* − 02	1.334*E* − 02	1.404*E* − 02	1.645*E* − 02	2.080*E* − 02	2.774*E* − 02	3.539*E* − 02	3.846*E* − 02	5.012*E* − 02	5.104*E* − 02
0.850	6.548*E* − 02	6.438*E* − 02	6.727*E* − 02	6.918*E* − 02	7.424*E* − 02	8.088*E* − 02	8.965*E* − 02	9.290*E* − 02	1.053*E* − 01	1.098*E* − 01
1.200	1.890*E* − 01	1.853*E* − 01	1.938*E* − 01	1.922*E* − 01	1.890*E* − 01	1.864*E* − 01	1.832*E* − 01	1.819*E* − 01	1.762*E* − 01	1.869*E* − 01
1.650	3.249*E* − 01	3.185*E* − 01	3.330*E* − 01	3.296*E* − 01	3.241*E* − 01	2.991*E* − 01	2.758*E* − 01	2.681*E* − 01	2.327*E* − 01	2.420*E* − 01
2.200	4.246*E* − 01	4.191*E* − 01	4.320*E* − 01	4.284*E* − 01	4.069*E* − 01	3.763*E* − 01	3.370*E* − 01	3.250*E* − 01	2.634*E* − 01	2.617*E* − 01
2.875	4.435*E* − 01	4.462*E* − 01	4.421*E* − 01	4.385*E* − 01	4.107*E* − 01	3.857*E* − 01	3.505*E* − 01	3.409*E* − 01	2.769*E* − 01	2.550*E* − 01
3.675	3.821*E* − 01	3.988*E* − 01	3.643*E* − 01	3.513*E* − 01	3.154*E* − 01	3.111*E* − 01	2.906*E* − 01	2.857*E* − 01	2.444*E* − 01	2.167*E* − 01
4.625	3.206*E* − 01	3.572*E* − 01	2.789*E* − 01	2.435*E* − 01	2.208*E* − 01	2.178*E* − 01	2.140*E* − 01	2.125*E* − 01	2.059*E* − 01	1.867*E* − 01
6.075	1.446*E* − 01	1.300*E* − 01	1.288*E* − 01	1.136*E* − 01	1.007*E* − 01	6.662*E* − 02	6.111*E* − 02	1.530*E* − 02	1.482*E* − 02	1.472*E* − 02

**Fig. 5:**
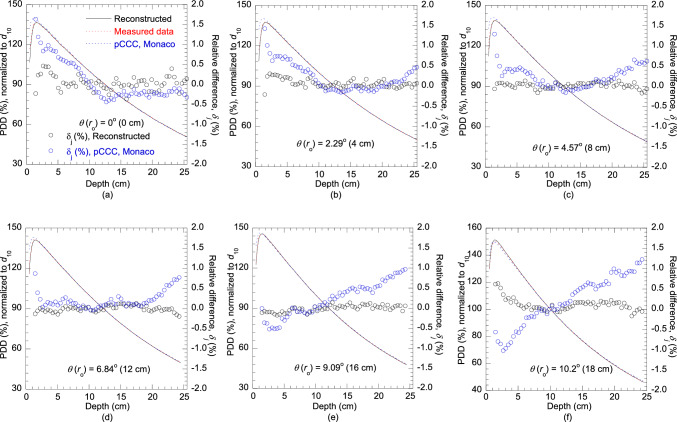
Comparisons of the PDDs reconstructed in this study and computed by the Monaco TPS with measured PDDs along the off-axis angles *θ* of 0°, 2.29°, 4.57°, 6.84°, 9.09°, and 10.2° for a 6 MV photon beam in a 40 cm × 40 cm field and SSD of 100 cm

Figure 5 compares the PDDs calculated by the Monaco TPS, reconstructed in this study, and measured at ${r}_{0}$ of 0, 4, 8, 12, 16 and 18 cm along *θ* of 0°, 2.29°, 4.57°, 6.84°, 9.09°, and 10.2°. As mentioned in Sect. 2.1, since the build-up region was not considered in the PDD reconstruction process, corresponding relative differences *δ*_*j*_ (%) from the *d*_max_ (1.5 cm) were included in each graph. All reconstructed PDDs favorably agreed with the measured PDDs, with relative differences within ± 0.5%, but the PDDs calculated by the Monaco TPS differed significantly from the measured PDDs. At the central-axis, the calculated PDD was higher than the measured PDD at shallower depths, with maximum relative difference exceeding 1%, while at deeper depths (> 10 cm), calculated PDD constantly remained lower. As the ${r}_{0}$ increased, the calculated PDDs at shallower depths gradually became lower than the measured PDDs, resulting in a maximum relative difference larger than –1% at ${r}_{0}$ of 18 cm. Consequently, the calculated PDDs at deeper depths gradually increased with ${r}_{0}$ and eventually exceeded the measured PDDs, with a maximum relative difference above 1% at ${r}_{0}$ of 18 cm.

**Fig. 6:**
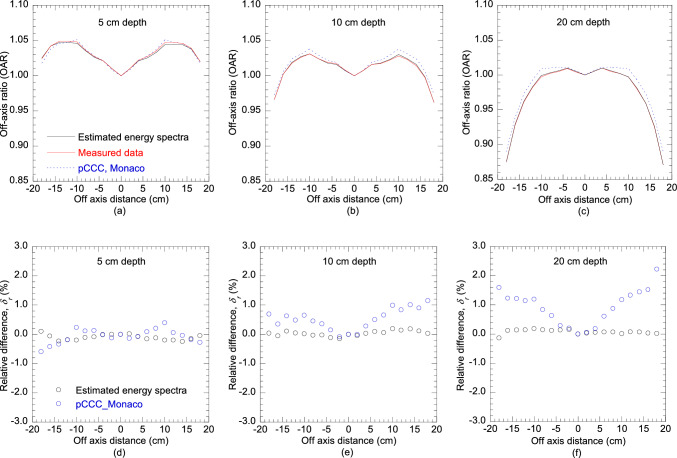
Comparisons of the OARs calculated using the estimated energy spectra, pCCC algorithm of the Monaco TPS, and measured at different depths: **a** 5 cm, **b** 10 cm, and **c** 20 cm; **d**–**f** Comparisons of their corresponding relative differences *δ*_*r*_ (%)

Figure 6a–c compare the OARs calculated by the Monaco TPS and the estimated energy spectra with measured OARs at depths of 5 cm, 10 cm, and 20 cm. The corresponding relative differences *δ*_*r*_ (%) are presented in Fig. 6d–f. The OARs calculated from the estimated energy spectra favorably agreed with the measured OARs (relative difference within ± 0.5%). In contrast, OARs calculated by the pCCC algorithm of the Monaco TPS deviated from the measured OARs, with relative differences exceeding + 1% at depths of 10 cm and 20 cm.

## Discussion

This study introduced a simple PDD reconstruction method for estimating energy spectra as functions of distance from the central-axis for the pCCC algorithm of the Monaco TPS. The reconstructed PDDs at various off-axis angles and the OARs calculated from the estimated energy spectra at different depths well agreed with the measured PDDs and OARs. The resultant relative differences were within ± 0.5%. Moreover, the energy spectra estimated at different radial distances showed noticeable off-axis softening, as expected (Fig. 4a).

The accuracy of the off-axis softening process of the Monaco TPS was also analyzed. The PDDs and OARs calculated by the Monaco TPS significantly differed from the measured PDDs and OARs, respectively, with relative differences exceeding ± 1%. The mean energy in the central-axis was estimated as 2.31 MeV in this study, which was comparatively higher than 2.24 MeV of Monaco. Owing to the lower mean energy of the Monaco TPS at the central-axis, the calculated PDD of the Monaco TPS was higher than the measured PDD at shallower depths and lower at deeper depths. However, as the radial distance increased, the PDDs gradually decreased at shallower depths and increased at deeper depths indicating the lack of off-axis softening (see Fig. 5). The relative differences exceeded ± 1% at a radial distance of 18 cm. According to these results, the energy spectra off-axis softening approximated by the Monaco TPS cannot accurately represent the actual off-axis softening. Therefore, estimation of precise energy spectra as functions of distance from the central-axis are required. To this end, we propose the reconstruction of PDDs at various off-axis angles as shown in this study for accurate dose calculation. The GRG optimization process employed in this study took only a few seconds to estimate each energy spectrum, thus ensuring that there is no increase in beam modeling time. Although the MDD calculations required for the PDD reconstruction are time-consuming, they can be precalculated and repeatedly employed for modeling the spectral distributions at any beam energy or linac. The MDDs calculated for this study, applicable for estimating the energy spectra for 4 MV and 6 MV, are given in Online Resource 1. Previously, in the Pinnacle TPS, energy spectra were considered as functions of radial distance, although they were indirectly estimated through an off-axis softening parameter, thus lacking precision [22]. With significant advancements in computational power in recent years, direct application of energy spectra, similar to the Pinnacle TPS approach, might be feasible without a notable increase in computing time. Alternatively, energy spectra-weighted point-energy deposition kernel can be applied.

## Conclusion

This study introduced a simple PDD reconstruction method for estimating energy spectra as functions of distance from the central-axis. The accuracy of the method was verified by comparing the PDDs and OARs obtained from the proposed and measurement approaches. The reconstructed PDDs and calculated OARs favorably agreed with the measured PDDs and OARs, with relative differences within ± 0.5%.

The uncertainties in the dose distributions calculated by the pCCC algorithm of the Monaco TPS, which result from inaccurate approximations of the energy spectra off-axis softening, were also investigated. The PDDs and OARs calculated by the Monaco TPS significantly differed from the measured PDDs and OARs, with resultant relative differences exceeding ± 1%. To minimize these uncertainties, precise estimation of energy spectrum not only at the central-axis but also at off-axis positions is required. The proposed PDD reconstruction method can estimate energy spectra as functions of distance from the central-axis, enabling accurate dose calculations.

## Supplementary Information

Below is the link to the electronic supplementary material.

Supplementary file1 (XLSX 328 KB)

## Data Availability

The MDDs (Gy/Incident fluence) calculated for this study are provided in the Online Resource 1.xlsx.
